# Uncovering the Anticancer Mechanisms of Chinese Herbal Medicine Formulas: Therapeutic Alternatives for Liver Cancer

**DOI:** 10.3389/fphar.2020.00293

**Published:** 2020-03-18

**Authors:** Feiyu Chen, Zhangfeng Zhong, Hor Yue Tan, Wei Guo, Cheng Zhang, Chi-wing Tan, Sha Li, Ning Wang, Yibin Feng

**Affiliations:** School of Chinese Medicine, Li Ka Shing Faculty of Medicine, The University of Hong Kong, Hong Kong, Hong Kong

**Keywords:** cancer, liver cancer, hepatocellular carcinoma, Chinese herbal medicine formula, alternative therapy

## Abstract

The potential values of Chinese herbal formulas in treating various diseases are well known. In addition to more than 2,000 years of history, herbal medicine is appreciated for its remarkable efficacy in a lot of cases, which warrants a role in public health care worldwide, especially in East Asian countries. Liver cancer is the second most fatal cancer across the world. Recent studies have extensively investigated the chemical profiles and pharmacological effects of Chinese herbal medicine formulas on liver cancer. Either through observational follow-up or experimental studies, multiple herbal formulas have benefits implicated in the management of liver cancer. However, complex composition of each formula imposes restrictions on promoting clinical practice and global recognition. Therefore, understanding the mode of action of Chinese herbal medicine formulas in depth may offer sufficient evidence for their clinical use. This review highlighted the chemical characteristics and molecular mechanisms of actions of prominent Chinese herbal medicine formulas and summarized the correlated findings on the potential use in liver cancer treatment. At last, the present progresses of Chinese herbal medicine formulas in the perspective of clinical trials are discussed.

## Introduction

Liver cancer is highly fatal, with an estimation of 841,000 new cases and 782,000 new deaths for liver cancer that occurred around the world in 2018 (Bray et al., 2018). In contrast to any other cancers, liver cancer incidence and mortality are both increasing at a faster pace by almost 3% per year (Siegel et al., 2017). As of now, liver transplantation, image-guided ablation, and chemoembolization are state-of-the-art therapeutic modalities in practice. Even so, Asian countries are still undergoing the rapidly increasing cancer burden due largely to immense scale of population as well as less abundant medical resources. Compared to the United States, China has relatively poorer survival rate, with 40% higher cancer-related death among patients diagnosed with cancer (Feng et al., 2019).

Various forms of complementary and alternative medicine have been studied and practiced to deal with cancers or ailments (Barnes et al., 2008). A wide spectrum of medicinal herbs as well as their natural constituents or extracts has been demonstrated to possess anticancer properties with involvement of possible mechanisms including cell cycle arrest, cell apoptosis induction, inflammation suppression, immune modification, and angiogenesis inhibition. For example, *Coptidis chinensis* Franch (Huanglian) extract and its main active compound, berberine, were revealed antineoplastic activity via inducing cell cycle arrest and cell apoptosis, as well as inhibiting metastasis and angiogenesis (Wang et al., 2015b), partly involving tumor suppressor p53 and miR-23a pathway (Wang et al., 2014). Moreover, targeting the hypoxia-inducible factor 1 signaling pathway is a key antiangiogenic mechanism behind cancer treatment with the formula Pien Tze Huang, as well as famous compounds such as curcumin, ginsenosides, and baicalein (Hong et al., 2019). Based on the fact that Danshensu is the major bioactive constituent of *Salvia miltiorrhiza* Bunge (Danshen), novel compounds were designed as multidrug resistance reversal agents, and Danshensu-tetramethylpyrazine conjugate was observed to overcome multidrug resistance via simultaneously inhibiting P-gp activity and regulating metabolic process (Zhou et al., 2019).

**Figure 1:**
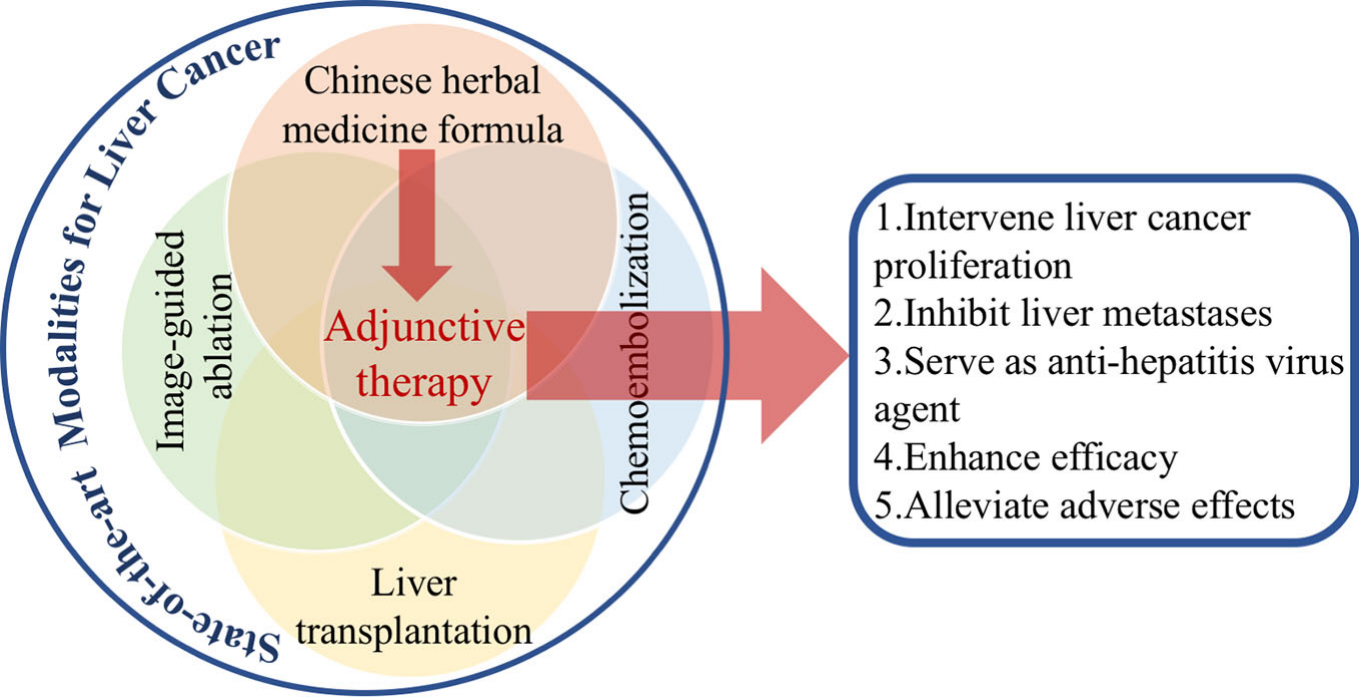
The roles of Chinese herbal medicine formulas in liver cancer treatment.

Nevertheless, with 2,000-year empirical evidence, Chinese medicine has always been characterized with holistic perspective and syndrome differentiation. Based on these principles of diagnosis and treatment, cancer is rather believed as the “cumulative toxicity” of internal organs that combines several patterns of syndrome (Liu et al., 2015). The application of herbal formulas is being well recognized for those multicomponents in confronting complexity of cancer. More so, formulas are favorable in cancer complications management, leading to less function impairment, pain alleviation, sleep improvement, and depression remission (Tao et al., 2015b). In the Chinese Pharmacopoeia (2015 edition), a total of 25 formulas are documented with antineoplastic efficacy, featuring with Qi invigorating, heat clearing and detoxifying, blood activating, and stasis removing, as well as phlegm removing (Huang et al., 2019). Among them, Qi tonifying and detoxification are two main strategies for liver cancer therapy. Combining herbal medicines with diverse functions could synergistically benefit cancer patients (**Figure 1**).

**Figure 2:**
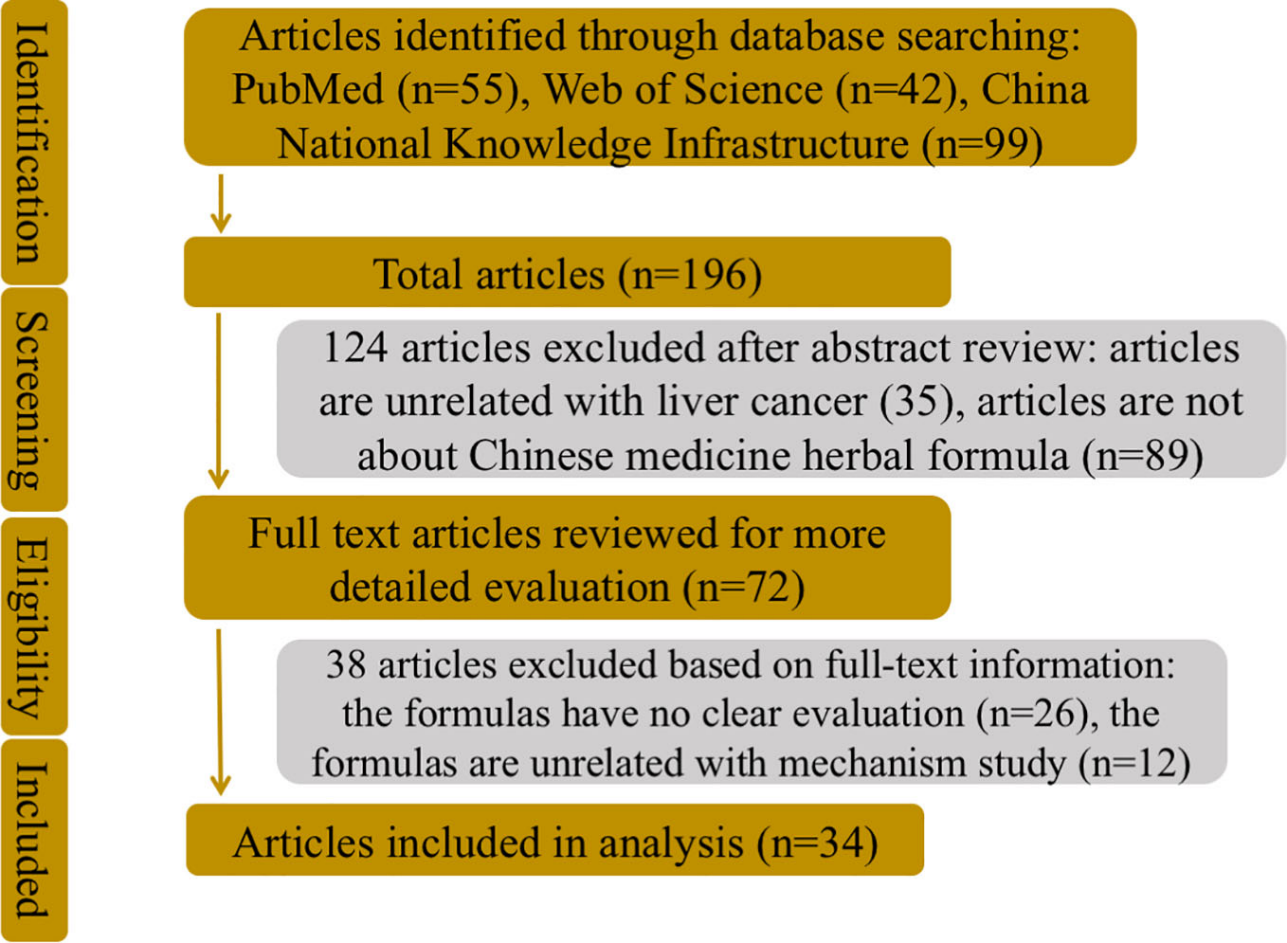
The flowchart of the literatures search strategy, inclusion and exclusion criteria.

To better understand molecular mechanisms whereby Chinese herbal medicine formulas exert their antineoplastic efficacies in liver cancer as well as current status of formulas in clinical practice, database retrieval was conducted in PubMed and Web of Science, as well as China National Knowledge Infrastructure, with terms including *liver cancer*, *hepatocellular carcinoma*, *Chinese medicine*, *herbal formula*, and *Chinese medicine herbal formula*, alone or in randomized combination (**Figure 2**). Publications in English and Chinese were both included (**Table 1**).

**Table 1:** Chinese herbal medicine formulas for liver cancer treatment.

Name of formula	Composition	Dosage or interval	Key findings	Mechanism of action	Reference
Compound *Astragalus* and *Salvia miltiorrhiza* extract	*Astragalus membranaceus Bunge*, *Salvia miltiorrhiza Bunge*	60, 120, or 240 mg/kg per day for 12–16 weeks	Repressed proliferation of hepatic stellate cells and HepG2 cells, inhibited tumor growth in HCC rats	MAPK (mitogen-activated protein kinases)–regulated TGF (transforming growth factor)-β/Smad signaling pathway	(Boye et al., 2015)
Ciji Hua'ai Baosheng granule	*Codonopsis pilosula* (*Franch*.) *Nannf*., *Astragalus membranaceus Bunge*, *Arisaema erubescens* (*Wall*.) *Schott*, *Ostreae Concha*, *Ganoderma*, *Sparganium stoloniferum* (*Buch.-Ham. ex Graebn*.) *Buch.-Ham. ex Juz*., *Gleditsia sinensis Lam*., *Cullen corylifolium* (*L*.) *Medik*., *Citrus aurantium L*.	5.85, 2.93, or 1.46 g/mL per day for 10 days	Prolonged survival time of H22 mice model	Increasing serum IL-2 (interleukin 2), IFN-γ (Interferon-γ) and TNF-α, but decreased IL-6 levels in serum and tumor tissue	(Xi et al., 2014; Xi et al., 2018)
Dachaihu decoction	*Bupleurum chinense DC*., *Scutellariae baicalensis Georgi*, *Paeonia lactiflora Pall*., *Pinellia ternata* (*Thunb*.) *Makino*, *Citrus aurantium L*., *Zingiber officinale Roscoe*, *Ziziphus jujube Mill*.	50 mL/time twice a day (BID), for 5 days	Attenuated adverse reactions (fever, abdominal pain, , nausea and vomiting, insomnia) in HCC patients	NA	(Wang et al., 2019)
Dahuang Zhechong formula	*Rheium palmatum L*., *Prunus persica* (*L*.) *Batsch*, *Scutellariae baicalensis Georgi*, *Prunus armeniacae L*., *Inula helenium L*., *Eupolyphaga seu Steleophaga*, *Tabanus*, *Hirudo*, , *Holotrichia diomphalia Bates*, *Paeoniae lactiflora Pall*., *Glycyrrhiza uralensis Fisch*, *Rehmannia glutinosa* (*Gaertn*.) *DC*.	In types of pill and capsule for 12 weeks to 12 months	Reduced fibrosis markers in liver fibrosis patients with Hepatitis B virus, potential benefits for liver cancer patients	NA	(Zhang et al., 2013)
Danzhi Xiaoyao San	*Paeonia suffruticosa Andrews*, *Gardenia jasminoides J. Ellis*, *Bupleurum chinense DC*., *Angelica sinensis* (*Oliv*.) *Diels*, *Paeonia lactiflora Pall*., *Atractylodis Macrocephalae Koidz*, *Campsis grandiflora* (*Thunb*.) *K. Schum*., *Glycyrrhiza uralensis Fisch*.	250 mL/time BID, for 15 days	Tumor inhibition and reduced incidence of adverse reactions in HCC patients	NA	(Zhao et al., 2011)
Fuzheng Jiedu Tongluo Fang	*Curcuma longa L*., *Lobelia chinensis Lour*., *Scutellaria barbata D. Don*, *Reynoutria japonica Houtt*., *Atractylodis Macrocephalae Koidz*, *Astragalus membranaceus Bunge*, *Angelica sinensis* (*Oliv*.) *Diels*, *Spatholobus suberectus Dunn*, *Cinnamomum cassia* (*L*.) *J. Presl*	2.65 mg/mL	Inhibited invasion of HepG2 cells.	MMP-2 (matrix metalloproteinase 2) and MMP-9 downregulation	(Zhang et al., 2017)
Fuzheng Kang'ai Formula	*Astragalus membranaceus Bunge*, *Ligustrum lucidum W. T. Aiton*, *Curcuma longa L*., *Actinidia arguta* (*Sieb. & Zucc*) *Planch. ex Miq*., *Salvia miltiorrhiza Bunge*	13.6 µmol/L	Blocked invasion and migration capacities in HepG2 cells	Reversing epithelial-to-mesenchymal transition	(Fang et al., 2019)
Fuzheng Yiliu decoction	*Sparganium emersum Rehmann*, *Curcuma longa L*., *Solanum nigrum L*., *Solanum lyratum Thunb*., *Scutellariae baicalensis Georgi*, *Hedyotis Diffusa Willd*, *Astragalus membranaceus Bunge*, *Angelica sinensis* (*Oliv*.) *Diels*, *Atractylodis Macrocephalae Koidz*	400 mg/mL	Inhibited tumor progression, invasion, and migration ability in MHC97-H cells	NA	(Peng et al., 2018)
Gansui Banxia Tang	*Euphobiae Kansui T. N. Liou ex S. B. Ho*, *Pinelliae ternata* (*Thunb*.) *Makino*, *Glycyrhiza glabra L*., *Paeonia lactiflora Pall*.	7.2–14.4 g/kg per day	Inhibited tumor growth in xenografted nude mice	Targeting Hsp90a (heat shock protein 90a), ATP1A1 (ATPase Na+/K+ transporting subunit α1) and STAT3 (Signal transducer and activator of transcription 3) proteins	(Zhang et al., 2014)
*Hedyotis diffusa* Willd. detoxification spleen prescription	*Hedyotis diffusa* Willd., *Atractylodis Macrocephalae Koidz*, *Codonopsis pilosula* (*Franch*.) *Nannf*., *Dioscorea japonica Thunb*., *Citrus aurantium L*., *Setaria italica* (*L*.) *P. Beauv*., *Glycyrrhiza uralensis Fisch*	NA	Alleviated the pain of patients and side effects, inhibited the proliferation of tumor cells in patients	NA	(Cai and Liao, 2017)
Huanglian Jiedu decoction	*Coptis chinensis Franch*., *Phellodendron chinense C. K. Schneid*., *Scutellaria baicalensis Georgi*., *Gardeniae jasminoides J. Ellis*.	25, 50, and 100 mg/kg, BID, for 3 weeks	Attenuated cell progression in HepG2, MHCC97L cells; suppressed xenografted growth of nude mice	Targeting eukaryotic elongation factor 2	(Wang et al., 2015a)
JDF granule	*Actinidia chinensis Planch*., *Salvia miltiorrhiza Bunge*, *Cremastra appendiculata* (*D. Don*) *Makino*, *Galli gigerii endothelium Corneum*	8 g/time BID	Prolonged survival of patients with unresectable HCC	NA	(Yu et al., 2009)
Jiedu Xiaozheng Yin	*Hedyotis diffusa* Willd., *Sophora flavescens Aiton*, *Pseudobulbus cremastrae* (*D. Don*) *Makino*, *Prunella vulgaris L*.	200 mg/mL	Inhibited cell growth of HepG2 cells	Cell arrest at G0/G1 phase via regulated expressions of cyclin D and cyclin E	(Cao et al., 2013)
Ka-mi-kae-kyuk-tang	*Benincasa hispida* (*Thunb*.) *Cogn*., *Bletilla striata* (*Thunb*.) *Rchb.f*., *Amana edulis* (*Miq*.) *Honda*, *Panax ginseng C. A. Mey*., *Vigna angularis* (*Willd*.) *Ohwi & H. Ohashi*, *Zanthoxylum piperitum* (*L*.) *DC*., *Patrinia villosa* (*Thunb*.) *Dufr*., *Astragalus membranaceus Bunge*, *Angelica sinensis* (*Oliv*.) *Diels*, *Achyranthes* BID*entata Blume*	3 times/week for 36 days	Anti–liver metastasis and invasiveness in C57BL/6 mice model	NA	(Lee et al., 2006)
KCT-01	*Artemisia capillaris Thunb*., *Sanguisorba officinalis L*., *Curcuma longa L*.	5,000 mg/kg per day	Cell proliferation reduced in HepG2 and HepG2.2.15 cells; anti-HBV in C57BL/6 mice	Reduction in TNF-α, IL-6, and MCP (methyl-accepting chemotaxis proteins) mRNA synthesis	(Kim et al., 2018)
Liujunzi Decoction	*Corydalis yanhusuo* (*Y. H. Chou & Chun C. Hsu*) *W. T. Wang ex Z.Y.Su & C.Y.Wu*, *Glycyrrhiza uralensis Fisch*, *Aucklandia costus Falc*., *Amomum villosum Lour*., *Pseudostellaria heterophylla* (*Miq*.) *Pax*, *Artemisia scoparia Waldst. & Kit*., *Atractylodis Macrocephalae Koidz*, *Pinellia ternata* (*Thunb*.) *Makino*, *Paeonia lactiflora Pall*., *Scutellariae baicalensis Georgi*, *Bupleurum chinense DC*.	BID for 9 days	Better scores in life quality and liver function (alanine aminotransferase, aspartate aminotransferase) in HCC patients	NA	(Zhang, 2019)
Pien Tze Huang	*Moschus*, *Bos taurus domesticus Gmelin*, *Agkistrodon halys* (*Pallas*)., *Panax notoginseng* (*Burkill*) *F. H. Chen*	234 mg/kg per day	Anti–liver metastasis	Inhibition of epithelial-to-mesenchymal transition; regulation of Hif-1 (hypoxia-inducible factor 1) pathway	(Lin et al., 2015; Hong et al., 2019)
PHY906	*Scutellariae baicalensis Georgi*, *Glycyrrhiza uralensis Fisch*, *Paeonia lactiflora Pall*., *Ziziphus jujube Mill*	800 mg BID	Median overall survival was 9.2 months in advanced HCC patients	Synergistically acting with chemotherapy	(Yen et al., 2009)
QHF (Q, Qingrejiedu; H, Huoxuehuayu; and F, Fuzhengguben)	*Panax ginseng C. A. Mey*., *Panax notoginseng* (*Burkill*) *F. H. Chen*.	20, 40, 80, 160, and 320 µg/mL	Inhibited migration and invasion in HepG2 cells	Inhibitory action in migration and invasion activities	(Tao et al., 2010; Chen et al., 2016)
Qingyihuaji formula	*Scutellaria barbata D. Don*, *Hedyotis fruticosa L*., *Amorphophallus kiusianus*, *Coix lacryma-jobi L*., *Gynostemma pentaphyllum* (*Thunb*.) *Makino*, *Geranium lucidum L*., *Amomum villosum Lour*.	NA	Anti–liver metastasis in nude mice	Reversing epithelial-to-mesenchymal transition	(Zhang et al., 2013)
San-Huang-Xie Xin-Tang	*Rheum palmatum L*., *Coptis chinensis Franch*., *Scutellaria baicalensis Georgi*.	3.66 mg/mL	Cell proliferation decreased in HepG2 cells	p53 signaling and DNA damage	(Cheng et al., 2008)
Shen-Ling-Bai-Zhu Powder	*Panax ginseng C. A. Mey*., *Poria cocos* (*Schw*.) *Wolf*, *Atractylodis Macrocephalae Koidz*., *Dioscoreae oppositifolia L*., *Lablab purpureus Subsp*., *Nelumbo nucifera Gaertn*., *Coix lacryma-jobi L*., *Amomum villosum Lour*., *Platycodon grandiflorus* (*Jacq*.) *A.DC*., *Glycyrrhiza uralensis Fisch*	0.075, 0.15, or 0.3 g/mL per day	Inhibited tumor growth and accelerated apoptosis in H22 mice model	Descending levels of tumor growth promoters signaling and apoptotic suppressor proteins	(Kim et al., 2018)
Shuangbai powder	*Phellodendron chinense C. K. Schneid*., *Platycladus orientalis* (*L*.) *Franco*, *Rheum palmatum L*., *Mentha canadensis L*., *Lycopus lucidus Turcz. ex Benth*.	BID for 7 days	Enhanced analgesic effect, reduced side effects, and improved patients' quality of life	NA	(Liu et al., 2016)
Shuihonghuazi formula	*Polygonum orientale L*., *Ophicalcitum Serpentine*, *Coix lacryma-jobi L*., *Imperata cylindrica* (*L*.) *Raeusch*.	757 mg/kg per day	Enhanced the organism immunity of cancer rats	Mediation of phosphatidylethanolamine *N*-methyltransferase (PEMT), lysophospholipase D, methylenetetrahydrofolate reductase (MTHFR) and lysophospholipase	(Bao et al., 2017)
Sijunzi decoction	*Panax ginseng C. A. Mey*., *Atractylodis Macrocephalae Koidz*, *Glycyrrhiza uralensis Fisch*	300 mL/day, for 2 years	Reduced tumor recurrence and increased survival rate in HCC patients	Regulation of T lymphocytes and natural killer cells	(Chen et al., 2017; Song and Wu, 2017)
Simo decoction	*Panax ginseng C. A. Mey*., *Areca catechu L*., *Aquilaria sinensis* (*Lour*.) *Spreng*., *Lindera aggregata* (*Sims*) *Kosterm*.	300 mL/day	Recovery of gastrointestinal function and reduced complications in HCC patients	NA	(Li, 2019)
Songyou Yin	*Salvia miltiorrhiza Bunge*, *Astragalus membranaceus Bunge*, *Lycium barbarum L*., *Crataegus pinnatifida Bunge*, *Trionyx sinensis Wiegmann*	4 g/kg per day	Tumor suppression and metastasis inhibition in C57BL/6 mice	Enhancing immunity (CD4, CD8), reduced serum TGF-β1 and CD4 + CD25 + Foxp3 + Treg (regulatory T cells) proportion in PBMC (peripheral blood mononuclear cell), spleen lymphocytes, and TIL (tumor-infiltrating lymphocytes)	(Zhang et al., 2016)
Weichang'an	*Pseudostellaria heterophylla* (*Miq*.) *Pax*, *Atractylodes macrocephala Koidz*., *Poria cocos* (*Schw*.) *Wolf*, *Glycyrrhiza uralensis Fisch*., *Sargentodoxa cuneata* (*Oliv*.) *Rehd. et Wilson*, *Prunella vulgaris L*.	0.5 mL/day	Cytotoxicity in HCT-116 cells; reduced metastasis in nude mice	Decreasing expressions of β-catenin and MMP-7	(Tao et al., 2015a)
Xiaotongsan	*Boswellia sacra Flueck*., *Myrrha Mitch*, *Corydalis DC*, *Melia azedarach L*., *Curcuma aromatica Salisb*., *Cinnamomum camphora* (*L*.) *J. Presl*, *Daemonorops draco BI*	Every day for 4 weeks	Relieved the pain of patients, decreased dosage of morphine	NA	(Shen et al., 2014)
Yanggan Huayu KangaiSan	*Astragalus membranaceus Bunge*, *Ziziphus jujube Mill*, *Atractylodis Macrocephalae Koidz*, *Dioscorea japonica Thunb*., *Paeonia lactiflora Pall*., *Glycyrrhiza uralensis Fisch*, *Praeparata Cum Melle*, *Pseudostellaria heterophylla* (*Miq*.) *Pax*, *Angelica sinensis* (*Oliv*.) *Diels*, *Lycium barbarum L*., *Corydalis yanhusuo* (*Y. H. Chou & Chun C. Hsu*) *W. T. Wang ex Z. Y. Su & C. Y. Wu*, *Ligustrum lucidum W. T. Aiton*, *Scutellaria barbata D. Don*, *Curcuma longa L*., *Citrus aurantium L*., *Cinnamomum cassia* (*L*.) *J. Presl*, *Sargentodoxa cuneata* (*Oliv*.) *Rehder & E. H. Wilson*, *Magnoliae Officinalis Cortex*, *Curcuma longa L*., *Bupleurum chinense DC*., *Salvia miltiorrhiza Bunge*, *Arisaema Cum Bile*, *Solanum Nigrum L*., *Pinellia ternata* (*Thunb*.) *Makino*, *Rheum palmatum L*., *Euphorbia kansui S. L. Liou ex S.B.Ho*.	12 g/time, thrice a day	Inhibited anchorage-independent growth and induced caspase-mediated anoikis in Bel-7402 cells	Reducing glutamate aminotransferase, aspartate aminotransferase, serum total bilirubin, serum α-fetoprotein	(Zhao et al., 2011)
Yanggan Jiedu Sangjie formula	*Ligustrum lucidum W. T. Aiton*, *Duchesnea indica* (*Andr*.) *Focke*, *Solanum nigrum L*., *Euphorbia helioscopia L*., *Ranunculus ternatus Thunb*., *Curcuma longa L*.	400 mg/mL	Inhibited anchorage-independent growth and induced caspase-mediated anoikis in Bel-7402 cells	ROS (reactive oxygen species) generation and PTK2 (PTK2 protein tyrosine kinase 2) downregulation	(Hu et al., 2018)
Yi Guan Jian	*Glehnia littoralis* (*A Gray*) *F. Schmidt ex Miq*., *Ophiopogon japonicus* (*Thunb*.) *Ker Gawl*., *Angelica archangelica L*., *Rehmannia glutinosa* (*Gaertn*.) *DC*., *Lycium barbarum L*., *Melia azedarach L*., *Reynoutria japonica Houtt*.	400 mg/mL	Cell proliferation decreased in Bel-7402 cells	Anoikis induction and phosphorylation of p38 MAPK	(Hu et al., 2011)
Zuo Jin Wan	*Coptis chinensis Franch*., *Tetradium ruticarpum* (*A. Juss*.) *T. G. Hartley*	19, 38, 76, 152, and 304 μg/mL	Inhibitory effects in SMMC-7721, BEL-7402, BEL-7404, HepG2 cells	Induction of mitochondria-dependent apoptosis	(Xu et al., 2014)
ZYD	*Phyllanthus urinaria L*., *Salvia miltiorrhiza Bunge*., *Arnebia guttata Bunge*	500, 1,000 μg/mL	Attenuated migratory and adhesion abilities in SMMC-7721	Anti–hepatitis B virus, deregulations of Snail, MMP-2 and MMP-9	(Guo et al., 2018)

## Chinese Herbal Medicine Formulas Intervene Liver Cancer Progression

According to the principles of Chinese medicine, liver cancer is being considered the cumulative toxicity of internal organs that combines several patterns of syndrome such as Qi-blood deficiency, phlegm stagnation, blood stasis, spleen deficiency, or damp-toxin condensation. However, there is a consensus in terms of the selection of therapeutic strategy, which is to invigorate Qi and eliminate toxic pathogens (Huang et al., 2019). As more than 5,000 medicinal herbs have been documented and practiced in Asia to date, as well as the same herbs usually present disparate functions in different formulas due to interactions with each other in different combinations, a valid and successful formula normally goes through several modifications in dosage or choice of herbs. As such, clarifying the molecular mechanisms underground the anticancer action of Chinese herbal medicine formulas might shed new light on liver cancer treatment.

### Ancient Herbal Formulas in Liver Cancer Treatment

Yi Guan Jian (YGJ) was established by Wei Zhixiu in Qing Dynasty. Its application in treating liver disease with liver-YIN deficiency has been well documented in Chinese medical monograph (Liu et al., 2005). With consideration that hepatoma generation may be attributed to liver-YIN insufficiency (Wu et al., 2007), YGJ has long been an optimal formula for liver cancer. Yet its tumor suppression activity is not as stable and sustained as anticipated; researchers optimized the original prescription by changing dosages of some herbs. After exposure of hepatoma Bel-7402 cells to modified YGJ, considerable decline in cell proliferation was observed, and the inhibitory action was further reported to be correlated with the induction of anoikis and p38 phosphorylation (Hu et al., 2011). The formula named San Huang Xie Xin Tang (SHXXT) is made up of three herbs, which are *Coptis chinensis* Franch (Huanglian), *Scutellaria baicalensis* Georgi (Huangqin), and *Rheum palmatum* L. (Dahuang). Their individual effects to restrain hepatocellular carcinoma have been demonstrated to modulate cell cycle or apoptotic profiles (Chang et al., 2002; Lin et al., 2004; Chan et al., 2006; Lu et al., 2007). Researchers analyzed gene profiles of HepG2 cells after exposure to SHXXT, indicating that SHXXT displayed antiproliferation pattern via p53 signaling and DNA damage (Cheng et al., 2008). Huanglian Jiedu decoction (HJD) is a canonical herbal formula that has been used for heat-damp–related diseases since its inception 1,300 years ago. Recently, its curative effect in several types of cancer is appreciated in the Asian community. Chinese medical practitioners have postulated HJD as a regimen for cancer treatment. Previous reports identified the tumor suppression of hepatocellular carcinoma after HJD administration, and further investigation indicated that eukaryotic elongation factor 2 might be a new target of the formula in attenuating cancer progression (Wang et al., 2015a). Zuo Jin Wan (ZJW) is a well-known drug pair composed of *C. chinensis* Franch and *Fructus rutaecarpa* Benth (*Wuzhuyu*), which was prescribed to remove heat and dampness from liver. Recent works have identified antineoplastic activity of ZJW in several types of tumors including gastric cancer (Tang et al., 2012), colorectal cancer (Sui et al., 2013), breast cancer (Du et al., 2013), and liver cancer (Chao et al., 2011; Chou et al., 2017). The mode of action underlying the antiproliferation effect of ZJW on these malignancies involved the induction of mitochondria-dependent apoptosis (Xu et al., 2014). Gansui Banxia Tang (GBT) is proposed and practiced dating back centuries by Zhang Zhongjing, who has been a Chinese medical sage of considerable standing. The formula has been used for treatment of effusion of pleural, peritoneal, pericardial, and cranial cavities and intestinal tuberculosis, as well as gastrointestinal inflammation. In modern times, it was shown that it may be effective in some malignancies and cancerous ascites, such as hepatocellular carcinoma and esophageal cancer (Xia, 1989; Song, 1993), yet the pharmacological mechanism behind the anticancer effect has not been fully elucidated. *Euphobiae kansui* S. L. Liou ex S.B.Ho (Gansui) and *Glycyrhiza glabra* L. (Daji), two herbs in GBT, which are traditionally regarded as prohibited combination in “18 antagonisms,” are, however, intriguingly included in the same formula. In order to illuminate the synergy as well as the action mechanism of the ingredients on hepatocellular carcinoma, Zhang and colleagues developed a comprehensive system of integrating disease-specific and drug-specific network, which revealed the associations of GBT ingredients with their putative targets, concurrently with hepatoma-related pathways. Moreover, further experiments showed that Hsp90a, ATP1A1, and STAT3 proteins might be targeting molecules in tumor repression (Zhang et al., 2014). Shuihonghuazi formula (SHHZF) is made up of four medical herbs including *Polygonum orientale* L. (Shuihonghuazi), *Ophicalcitum* Serpentine (Huaruishi), *Coix lacryma-jobi* L. (Yiyiren), and *Imperata cylindrica* L. Raeusch (Baimao). Over the past 30 years, growing clinical experiences have demonstrated considerable antineoplastic ability of SHHZF in liver cancer patients (Lee et al., 2008; Zhou et al., 2012). Since the mode of action remains obscure, researchers have adopted the metabolomics method to facilitate the understanding of metabolomic characteristics related to its function. The study indicated metabolic profiles involving mediation of phosphatidylethanolamine *N*-methyltransferase, lysophospholipase D, methylenetetrahydrofolate reductase, and lysophospholipase are responsible for the antitumor effect (Bao et al., 2017).

As we know, clinical employment of some formulas remains unsatisfactory due in part to extraction approach barrier. Successful discovery of artemisinin by Nobel Laureate Tu Youyou exemplifies the fact that inappropriate extraction method may undermine effectiveness. Screening for optimal extraction methods of herbal formulas may help to achieve better therapeutic outcome. Rather than the conventional method utilizing water to extract active constituents from herbal formulas, present works place much focus on extraction using different polarity solvents (Cao et al., 2013). Thus, Chinese herbal medicine formulas may deserve more attention in the field.

### Nascent Herbal Formulas in Liver Cancer Treatment

Beyond ancient formulas generally used in clinical practice, a wealth of nascent formulas has been used over the past few decades, and the relevant pharmacological actions have been investigated. Fuzheng Yiliu decoction (FYD) is a polyherbal formula consisting of Qi-blood–tonifying and heat toxin–clearing herbs. Human hepatocellular carcinoma HepG2 and MHC97H cells were exposed to FYD, and the findings showed inhibitory actions of FYD in tumor progression, invasion, and migration (Peng et al., 2018). Another formula composed of eight herbs is an empirical prescription initiated by a Chinese medical physician from Shaanxi province. The formula was demonstrated to reverse epithelial–mesenchymal transition in HepG2 cells, by which invasion and migration capacities were remarkably blocked (Fang et al., 2019). Matrix metallopeptidases (MMPs) are a family of proteases known to degrade extracellular matrix proteins, which in turn renders the reduction in cell adhesion, following the disruption of cellular processes. Upregulation of MMPs has been well documented in multiple types of tumor (Shay et al., 2015). Fuzheng Jiedu Tongluo formula is made up of 10 herbs and was reported to restrain invasion and migration abilities by decreasing the expression levels of MMP-2 and MMP-9 (Zhang et al., 2017). Based on clinical medications and related studies, Yanggan Jiedu Sangjie formula (YJSF) was established for hepatoma treatment. In present study, they evaluated the anticancer potential of YJSF on suspension human hepatocellular carcinoma Bel-7402 cells. YJSF inhibited anchorage-independent growth and induced caspase-mediated anoikis in Bel-7402 cells, which may be related to ROS generation and PTK2 downregulation (Hu et al., 2018). Several lines of evidence have identified the hepatoprotective role of *Astragalus membranaceus* Bunge (*Huangqi)* and *S. miltiorrhiza* Bunge (Roxas and Jurenka, 2007). The two herbs have seen positive efficacy in both *in vitro* and *in vivo* models of liver fibrosis and hepatoma (Chan et al., 2009; Chen et al., 2011). Researchers designed a synergized formula with active ingredients extracted from the two herbs using orthogonal studies and named it CASE (Yang et al., 2008). Mechanistically, CASE modulated TGF-β/Smad signaling pathway and inhibited TGF-β–specific target gene expression in liver fibrosis and hepatocellular carcinoma, whereas the amelioration of hepatoma phenotypic hallmarks was verified (Boye et al., 2015). Herbal formula QHF is composed of three types of herbs with characteristics of clear heating (Qingrejiedu, Q), blood circulation promoting (Huoxuehuayu, H), and energy consolidating (Fuzhengguben, F). Researchers optimized the composition ratios of the formula (Tao et al., 2010), followed by the underlying mechanism of the prescription in liver cancer being investigated, which showed dramatic improvement of QHF in inhibiting migration and invasion activities in hepatic carcinoma HepG2 cells (Chen et al., 2016).

## Chinese Herbal Medicine Formulas Inhibit Liver Metastases

Cancer metastasis is one of the major barriers to successful management of carcinomas. As a critical hub in the body, the liver is involved in numerous physiological processes (Trefts et al., 2017). With two large blood vessels connected to the liver, it is a main site of metastatic disease from gastrointestinal tract, particularly colonic, gastric, and pancreatic malignancies. Reciprocal interactions between tumor cells and adjacent normal cells are implicated in hepatic metastases. Drugs that reverse or attenuate such intercellular communication will be in favor of suppressing tumor metastasis. Because diverse cross-communications occur in cancer metastasis, Chinese medicine holds the holistic perspective that might be beneficial in metastasis suppression. In fact, some formulas have been investigated in dealing with liver metastasis. A well-known formula named Pien Tze Huang (PZH) has been demonstrated effective in the management of several types of tumors (Wan et al., 2017; Chen et al., 2018). Recent work reported that PZH could not only repress colorectal tumor growth, but also exert anti–liver metastasis through the inhibition of epithelial-to-mesenchymal transition (Lin et al., 2015). Moreover, upregulated miR-16 expression was observed in its anti–hepatocellular carcinoma activity (Qi et al., 2017). Herbal medicine is popular not only in China, but also in Korea with its development based on Chinese medicine. Ka-mi-kae-kyuk-tang (KMKKT) is a formula that comprises 10 oriental herbs. Korean researchers reported with bench experiments and preclinical trials that KMKKT inhibited invasiveness of mouse colon cancer 26-L5 cells, and liver metastasis was less likely to occur in mice model (Lee et al., 2006). Weichang'an (WCA) is a herbal formula prescribed by practitioners, and the principal functions are spleen invigorating and heat clearing. Previous efforts have identified that patients with gastric (Zhao et al., 2010; Xu et al., 2013) or colon cancer (Gu et al., 2006) could benefit from WCA, whereas recent work showed positive efficacy of WCA in colorectal tumor with hepatic metastasis, in which decreased expressions of β-catenin and MMP-7 may be involved in the inhibitory action (Tao et al., 2015a). Pancreatic cancer is a common digestive system disease. A seven-herb formula named QYHJ has been used to treat pancreatic cancer, and patients receiving it reported prolonged survival time. Several scholars established the model of pancreatic cancer with liver metastasis using nude mice and found that QYHJ suppressed liver metastasis from pancreatic tumor, at least by reversing epithelial-to-mesenchymal transition (Zhang et al., 2013).

## Chinese Herbal Medicine Formulas Serve as Antiviral Agents to Prevent Liver Cancer

The infection of hepatitis viruses accounts for the majority of liver cancer occurrence. The most predominant contributor to this high burden is the dissemination of chronic infections with hepatitis B virus (HBV) and hepatitis C virus. Infection of hepatitis viruses can progress toward liver cirrhosis, which is tightly associated with hepatocellular carcinoma (Feng et al., 2019).

Hepatocellular carcinoma is characterized as the most prevalent cancer in China in recent decades. Recent decline in incidence has been partly attributed to effective prevention and suppression of hepatitis viruses (Sherman, 2008). Chinese medicine has long been used to diminish viruses and bacteria, which are risk factors for many ailments and diseases. On account of effective and multitargeting actions against viral infections, herbal formulas are lately receiving increasing attention. Dahuang Zhechong formula (DZF) has been used for the treatment of chronic hepatitis B, but its efficacy in liver fibrosis is conflicting. For this purpose, researchers made a meta-analysis for the effect of DZF on liver fibrosis, suggesting that DZF as adjuvant treatment could reverse liver fibrosis in patients with HBV infection, and robust conclusion was reached that antifibrotic effect might be a potential benefit of DZT. Nevertheless, for long-term clinical use in liver cancer patients with HBV infection, more investigations should be carried out in laboratory and clinical trials (Wei et al., 2015). The herbal formula has capacity of being anti-HBV, namely, ZYD. Its effect on the biological behavior of liver cancer was evaluated using hepatocellular carcinoma SMMC-7721 cells, displaying that ZYD could considerably attenuate migratory and adhesion abilities of hepatoma cells by a dose-dependent manner. Further investigation demonstrated the deregulations of Snail, MMP-2, and MMP-9 expression at nucleic acid level (Guo et al.). Recent work from a Korean group newly developed an oriental formula, KCT-01, which is extracted from *Artemisia capillaris* Thunb (Yinchen), *Sanguisorba officinalis* L. (Diyu), and *Curcuma longa* L. (Jianghuang). They reported that KCT-01 is able to suppress HBV replication and inflammatory cytokine production with low risk of toxicity either in cell or in animal models, indicating the antiviral potential of using KCT-01 alone or in combination with entecavir (Kim et al., 2018).

Collectively, there is currently no eradication cure for hepatitis viruses because of the characteristic obstacle that viral minichromosome covalently closed circular DNA persistently exists in infected subjects (Charre et al., 2019). To some extent, Chinese herbal medicine formulas represent an alternative choice for antiviral therapy. In this situation, a range of liver cancer incidence could possibly be averted.

## Complementary Therapy for Liver Cancer Management

To our knowledge, curative outcome of current advanced therapeutic modalities including liver transplantation, image-guided ablation, and chemoembolization remains suboptimal in liver cancer patients due partly to multiple side effects. In this case, Chinese medicine herbal formulas have gained increasing attention because of fewer adverse effects and less toxicity to adjacent cells or tissues (Li et al., 2019; Zeng et al., 2019). Herein, several relevant examples are illustrated to show adjunctive and complementary roles of formulas in the treatment of malignancies. Shen-Ling-Bai-Zhu powder (SLBZP) is a classic herbal remedy that has been used in the management of gastrointestinal carcinoma for a long period of time. A comprehensive analysis was conducted to determine the precise role of SLBZP in hepatocellular carcinoma, which identified the antitumor property of the formula in hepatoma. More than that, compared to patients simply receiving chemotherapy, hepatoma subjects synchronously undertaking the formula reported better curative outcome, indicating the therapeutic merit of SLBZP coupled with chemotherapy for hepatoma. The mechanism behind involved descending levels of tumor growth promoters and apoptotic suppressor proteins (Xi et al., 2016). JDF granule comprised detoxifying endotoxic herbs and has been commonly used in Chinese clinics, and some compositions of the formula have demonstrated cytotoxic activity against several hepatoma cells such as BEL-7402 and SMMC-7721 (Xu et al., 2010). As hepatocellular carcinoma is an intricate disease with multivariate etiology, a synergistic strategy that Chinese herbal medicine is combined with modern therapeutic modalities has acquired extensive attention. Lately, a retrospective case study was performed to analyze the combinate employment of transcatheter arterial chemoembolization (TACE) with JDF for hepatocellular carcinoma therapy. The conclusion could be made that JDF granule considerably improved the prognosis of hepatocellular carcinoma patients undertaking TACE (Yu et al., 2009). PHY906 is a formula that has long been used for 1,800 years to treat distressing conditions of the gastrointestinal disorder. In the recent past, preclinical and early-phase clinical trials of PHY906 coupled with chemotherapy in patients with advanced hepatocellular carcinoma (Yen et al., 2009), pancreatic cancer and other gastrointestinal malignancies (Saif et al., 2010) have yielded promising results. Ciji Hua'ai Baosheng granule (CHBG) is an empirical formula originated by a Chinese medical practitioner from Fujian. The herbal compositions possess multiple capacities of reinforcing Qi, removing blood stasis, and dissipating phlegm, which have been confirmed to effectively alleviate symptoms of cancer patients. The group investigated the effect of CHBG on general health and survival time in H22 mice model with chemotherapy treatment, showing that CHBG obviously contributed to survival time of mice bearing subcutaneous transplanted tumor or ascitic tumor (Xi et al., 2014). One step further, CHBG was identified as complementary therapy for patients undertaking chemotherapy by improving immune function and attenuating side effects (Xi et al., 2018). It is well known that exercise is favorable in overcoming and preventing ailments and diseases; the combined application of exercise and herbal formulas has not yet been largely studied. Intriguingly, researchers investigated the possibility of Songyou Yin (SYY) in parallel with swimming in liver cancer treatment. SYY is a five-herb formula that was demonstrated to exert enhanced effect on tumor suppression and metastasis inhibition. Combined use of SYY and swimming exercise showed protective effects against liver cancer in animal models (Zhang et al., 2016).

## Clinical Trials of Chinese Herbal Medicine Formula Offer Options for Liver Cancer Treatment

Extensive laboratory experiments have been conducted to explore the mechanisms of action by herbal formulas in liver cancer management. Nevertheless, herbal formulas have not yet been incorporated into conventional health care due to a series of obstacles, such as safety concerns, quality control of herbs, and evidence from clinical trials. Systematic and rigorous clinical evaluation are essential to transform oriental herbal practices into evidence-based prescriptions, which could provide an insight into the application of herbal formulas in the management of hepatic cancer (Yu et al., 2009; Du et al., 2010).

As stated above, in contrast to classical herbal formulas, growing nascent formulas have been introduced and prescribed by Chinese medicine practitioners. To observe clinical effectiveness of Yanggan Huayu KangaiSan (YHKS) on advanced primary liver cancer, 25 patients were recruited and orally received the formula for two courses. After treatment, glutamate aminotransferase, aspartate aminotransferase, serum total bilirubin, serum α-fetoprotein descended obviously; other clinical complications including pain relief was improved, implying that YHKS could improve life quality of patients (Zhao et al., 2011). To investigate the clinical efficacy of Danzhi Xiaoyao San (DXS) plus ablation on liver tumor, a study comparing microwave ablation treatment with combinate use of ablation and DXS found significant differences. Within 80 cases, patients who took oral DXS reported reduced α-fetoprotein, as well as less incidence of adverse reactions (Zhu et al., 2017). Transcatheter hepatic arterial chemoembolization is one of the efficient therapeutic avenues for hepatic tumor, yet postoperative syndrome is a tough problem in most cases. Researchers recruited 60 patients with hepatic cancer, who had been diagnosed as postoperative syndrome prior to recruiting. After receiving Dachaihu decoction, a premier choice by medical sage Zhang Zhongjing for soothing liver and eliminating pathogens, adverse reactions including fever, abdominal pain, nausea and vomiting, and insomnia were found alleviated, which warrant further investigations and clinical practices (Wang et al., 2019). Around the same time, another study reported the curative outcome of combinatory employment of Dachaihu decoction and another canonical formula Liujunzi decoction, in the management of postoperative syndrome. The finding showed that patients who received combinatory formulas had better scores in life quality and liver function (Zhang, 2019). In addition, a recent work reported Sijunzi decoction, a tonic prescription documented in “Prescriptions People's Welfare Pharmacy,” could improve immune function of patients who received TACE as well as relieve their adverse reactions (Song and Wu, 2017).

Despite surgery is an effective modality for hepatic cancer patients with no extrahepatic metastasis, the postoperative surgery still negatively influences patients' life quality. Scholars recruited 128 postoperative patients, and half of the cases received Sijunzi decoction, while the remaining received placebo. They found that the recurrence rate in the treated group was reduced compared to placebo group, and the mechanism possibly involved regulation of T lymphocytes and natural killer cells (Chen et al., 2017). Interestingly, beyond combinatory use of a couple of formulas, Tui-na (Chinese massage) was also employed with classic formulas. Simo decoction in combination with acupoint massage exerted significant therapeutic efficacy in recovery of gastrointestinal function of primary liver cancer patients (Li, 2019).

Based on clinical knowledge, cancer patients are prevalently living with cancer pain, leading to limitations in daily activity. Recognizing cancer-induced pain and initiating specialist management are important for patients' welfare. *Hedyotis diffusa* Willd. is the principal component of the formula with abilities of clearing heat and detoxicating, promoting circulation, and removing stasis. In a recent observational study of 80 patients with cancer, patients who received *H. diffusa* Willd. Detoxification Spleen Prescription (HDSP) were found to have lower incidence of pain (Cai and Liao, 2017). Shuangbai powder has been an option in promoting blood circulation, removing blood stasis, reducing swelling, and relieving pain; current advice is therefore to use Shuangbai powder in cancer pain treatment. However, extensive evidence from clinics indicated modified Shuangbai powder was preferred when the original formula failed to relieve pain. An observational study of 90 patients showed that those applied modified Shuangbai powder reported consistent pain relief in comparison with patients applying placebo (Liu et al., 2016). There are many analgesics of first-line management, and pain relief could be observed immediately, but the effectiveness could not last for a longer time; also, adverse effects have been reported such as gastrointestinal damage (Ruchita et al., 2017). Xiaotongsan, an empirical formula for external use, was practiced for years by practitioners to treat cancer-induced pain. It could enhance efficacy of conventional drug morphine through multiple targets including improvement of immune function and repression of metastasis. That said, herbal formulas could be employed coupled with first-line management of refractory pain triggered by malignant cancer (Shen et al., 2014). Taken together, on the basis of laboratory and clinical evidence-based investigations, Chinese herbal medicine formulas might have potential as latent therapeutic alternatives for liver cancer treatment.

## Conclusion

Extensive evidence has highlighted the clinical application of Chinese herbal medicine formulas in cancer therapy. For liver cancer treatment, Chinese herbal medicine formulas improve the curative outcome whereby multicomponent and thereby multitarget against complex symptoms in liver cancer. Moreover, because current chemotherapy or radiotherapy leads to dose-limiting toxicities and substantial side effects, various functions as well as modifiable herbal compositions of the formula ensure synergistic effects and even fewer side effects. However, complexed and modifiable herbal constituents are exactly the major hurdle to clarify the underlying molecular mechanisms. On the other hand, there are cases that Chinese herbs may be harmful to the human body and cause serious toxicity when taken excessively or under inappropriate circumstances. It is also the complexed and unprecise constituents that have to take the large blame for incidences. Therefore, further optimization and large-scale validation are always imperative in order to improve the precision and safety of Chinese herbal medicine formulas used in liver cancer management. Attempts to develop herbal formulas into stable and potent modalities may offer options of considerable merit for global health care.

## Author Contributions

FC and ZZ retrieved the data and draft the manuscript. HT, WG, and CZ retrieved the data. C-WT, SL, and NW revised the manuscript. YF initiated the idea and drafted the manuscript.

## Funding

This research was partially supported by the Research Council of the University of Hong Kong (project codes: 104004092 and 104004460), Wong's donation (project code: 200006276), a donation from the Gaia Family Trust of New Zealand (project code: 200007008), the Research Grants Committee (RGC) of Hong Kong, HKSAR (Project Codes: 740608, 766211, 17152116 and 17121419) and Health and Medical Research Fund (Project code: 16172751).

## Conflict of Interest

The authors declare that the research was conducted in the absence of any commercial or financial relationships that could be construed as a potential conflict of interest.
